# The HEARTS partner forum—supporting implementation of HEARTS to treat and control hypertension

**DOI:** 10.3389/fpubh.2023.1146441

**Published:** 2023-07-24

**Authors:** Taskeen Khan, Andrew E. Moran, Pablo Perel, Paul K. Whelton, Michael Brainin, Valery Feigin, Deliana Kostova, Patricia Richter, Pedro Ordunez, Anselm Hennis, Daniel T. Lackland, Slim Slama, Daniel Pineiro, Sheila Martins, Bryan Williams, Leonard Hofstra, Renu Garg, Bente Mikkelsen

**Affiliations:** ^1^Department of Non-communicable Diseases, World Health Organization, Geneva, Switzerland; ^2^Resolve to Save Lives, New York, NY, United States; ^3^Department of Medicine, Columbia University, New York, NY, United States; ^4^Centre for Global Chronic Conditions, Department of Non-communicable Disease Epidemiology, London School of Hygiene & Tropical Medicine, London, United Kingdom; ^5^World Heart Federation, Geneva, Switzerland; ^6^Department of Epidemiology, Tulane University School of Public Health and Tropical Medicine, New Orleans, LA, United States; ^7^World Hypertension League, New Orleans, LA, United States; ^8^Department of Clinical Neurology, Danube University, Krems, Austria; ^9^World Stroke Organization, Geneva, Switzerland; ^10^National Institute for Stroke and Applied Neurosciences, Faculty of Health and Environmental Sciences, Auckland University of Technology, Auckland, New Zealand; ^11^Division of Global Health Protection, Center for Global Health, Centers for Disease Control and Prevention, Non-communicable Diseases and Mental Health, Atlanta, GA, United States; ^12^Pan American Health Organization/World Health Organization, Washington, DC, United States; ^13^Division of Translational Neurosciences and Population Studies, Department of Neurology, Medical University of South Carolina, Charleston, SC, United States; ^14^Universidad de Buenos Aires, Buenos Aires, Argentina; ^15^Universidade Federal of Rio Grande do Sul, Porto Alegre, Brazil; ^16^International Society of Hypertension, Essex, United Kingdom; ^17^Amsterdam UMC—Vrije Universiteit Amsterdam, Amsterdam, Netherlands

**Keywords:** hypertension, global health, cardiovascular diseases, partnerships, implementation, primary healthcare, health systems, universal health coverage

## Abstract

Cardiovascular diseases (CVD), principally ischemic heart disease (IHD) and stroke, are the leading causes of death (18. 6 million deaths annually) and disability (393 million disability-adjusted life-years lost annually), worldwide. High blood pressure is the most important preventable risk factor for CVD and deaths, worldwide (10.8 million deaths annually). In 2016, the World Health Organization (WHO) and the United States Centers for Disease Control (CDC) launched the Global Hearts initiative to support governments in their quest to prevent and control CVD. HEARTS is the core technical package of the initiative and takes a public health approach to treating hypertension and other CVD risk factors at the primary health care level. The HEARTS Partner Forum, led by WHO, brings together the following 11 partner organizations: American Heart Association (AHA), Center for Chronic Disease Control (CCDC), International Society of Hypertension (ISH), International Society of Nephrology (ISN), Pan American Health Organization (PAHO), Resolve to Save Lives (RTSL), US CDC, World Hypertension League (WHL), World Heart Federation (WHF) and World Stroke Organization (WSO). The partners support countries in their implementation of the HEARTS technical package in various ways, including providing technical expertise, catalytic funding, capacity building and evidence generation and dissemination. HEARTS has demonstrated the feasibility and acceptability of a public health approach, with more than seven million people already on treatment for hypertension using a simple, algorithmic HEARTS approach. Additionally, HEARTS has demonstrated the feasibility of using hypertension as a pathfinder to universal health coverage and should be a key intervention of all basic benefit packages. The partner forum continues to find ways to expand support and reinvigorate enthusiasm and attention on preventing CVD. Proposed future HEARTS Partner Forum activities are related to more concrete information sharing between partners and among countries, expanded areas of partner synergy, support for implementation, capacity building, and advocacy with country ministries of health, professional societies, academy and civil societies organizations. Advancing toward the shared goals of the HEARTS partners will require a more formal, structured approach to the forum and include goals, targets and published reports. In this way, the HEARTS Partner Forum will mirror successful global partnerships on communicable diseases and assist countries in reducing CVD mortality and achieving global sustainable development goals (SDGs).

## Introduction

During the last three decades (1990–2019), cardiovascular disease (CVD), including ischemic heart disease (IHD) and stroke, has been the leading cause of death and disability, globally. In 2019, CVD accounted for 18.6 million deaths and 393.1 million disability-adjusted years of life lost (DALYs) with over three quarters of the IHD and stroke related deaths occurring in low- and middle-income countries. Although the overall rate of age-adjusted CVD deaths and DALYs declined between 1990 and 2019 (death rates—by 32%; DALYs—by 31%), the absolute burden of disease resulting from CVD increased by about 50% (deaths—by 54% and DALYs—by 40% and prevalent cases of CVD nearly doubled from 271 million (257–285) in 1990 to 523 million (497–550) in 2019, with a trend toward increasing death and DALYs rates from 2014 onwards in people younger than 70 years (1). These trends are especially concerning in certain regions for example in the Americas, where, after decades of sustained reduction, CVD mortality has begun to slow down and even increase (2).

High systolic blood pressure (systolic BP ≥115 mm Hg) is the single most important risk factor of death from all causes (19.2% of all deaths in 2019 were attributed to high systolic BP), and particularly from CVDs (53.7%) of deaths due to CVDs in 2019 were attributed to high SBP) (1). There are ~1.28 billion adults with an average systolic BP >140 mm Hg, a common threshold for treatment with antihypertensive medications. Globally, only 23% of adult women and 18% of adult men with hypertension (systolic BP >140 mm Hg) have their systolic blood pressure controlled to <140 mm Hg (3).

The Sustainable Development Goal (SDG) target 3.4 is to reduce premature mortality from non-communicable diseases (NCDs) by one third by 2030 (compared to 2015 data). Premature mortality from NCDs is decreasing overall globally however it is far too slow to actually achieve target 3.4 (4). In the Lancet countdown series, Watkins et al. presented modeled effects of potential interventions needed to be implemented by countries to meet SDG 3.4. They concluded CVD interventions featured prominently in this modeling because it includes the two biggest drivers of mortality (IHD and stroke) and because if implemented the effects of these interventions will demonstrate impact on mortality in the shorter term. The main interventions for CVDs demonstrated to have this impact are CVD primary prevention (including treatment for hypertension), aspirin for suspected acute coronary syndrome and treatment for heart failure (5).

A combination of population approaches to reduce tobacco, sodium consumption and improve diet as well as better management of conditions is required to achieve the SDG target (6, 7). In 2016, the World Health Organization (WHO) and the United States Centers for Disease Control (CDC) launched the Global Hearts initiative,^1^ including the HEARTS technical package for CVDs management in primary health care,^2^ to support governments in their goal to strengthen the prevention and control of CVD and to facilitate achievements of the UN health-related SDG goals. Over time, the initiative expanded to include more organizational support in the form of a HEARTS partner forum. The purpose of this paper is to describe the HEARTS partner forum, outline it's role and demonstrate how the forum has influenced the implementation of HEARTS to treat and control hypertension as a population wide approach. This is done by reviewing the HEARTS strategic approach to improving CVD health, its advantages, feasibility and progress of implementation and further outlines future directions for HEARTS and the HEARTS partner forum.

## The HEARTS partner forum

**Figure 1:**
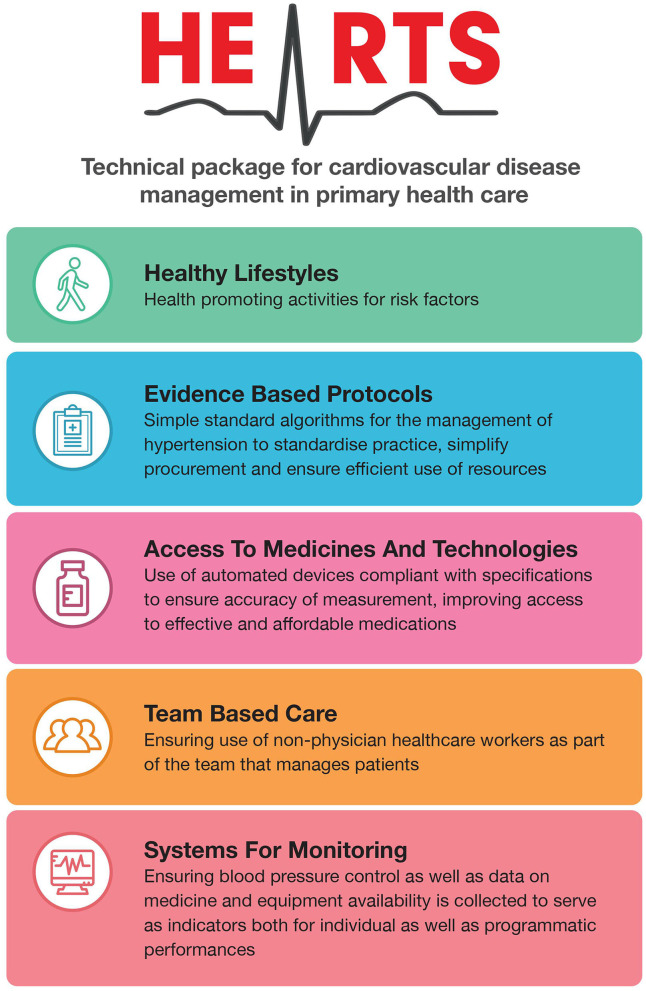
Adaptation of HEARTS technical package to manage hypertension.

The HEARTS technical package was launched in 2018 as the core technical package of the Global Hearts Initiative. The package is the public health approach to managing hypertension and other CVD risk factors such as dyslipidaemia at the primary healthcare level with the aim to be incorporated into universal health coverage benefit packages. Six modules and an implementation guide make up the package. The modules are Healthy Lifestyles, Evidence-Based Protocols, Access to Medicines and Technologies, Risk based management, Team based care and Systems for Monitoring. The package was developed through technical working group meetings, external consultation, and peer review over a period of 2 years. Once finalized by WHO, the package was adapted (Figure 1) and endorsed by 12 partner organizations namely, American Heart Association (AHA), Center for Chronic Disease Control (CCDC), International Diabetes Federation, International Society of Hypertension (ISH), International Society of Nephrology (ISN), Pan American Health Organization (PAHO), Resolve to Save Lives (RTSL), US CDC, World Hypertension League (WHL), World Heart Federation (WHF) and World Stroke Organization (WSO) to manage hypertension.

This partnership was formalized as the HEARTS partner forum, a technical forum that supports the implementation of the HEARTS technical package with a current focus on managing hypertension in primary healthcare. The partners provide catalytic funding, technical support, capacity building, evidence generation, communication and advocacy to countries implementing HEARTS (Figure 1). Underpinning the partnership is the normative guidance created by WHO which is then endorsed by the partners rendering to better downstream support, advocacy, communication, and dissemination of the recommendations.

### Other global health partnerships

There are various global health partnerships concentrating on different disease areas usually with underlying goals of elimination or eradication lending themselves more to the historical approach of addressing communicable diseases.

The most widely known mechanism is the Global Fund which is a large-scale movement that brings together world leaders, communities, civil society, health workers and the private sector to raise and invest funds to defeat Human-Immunodeficiency-Virus (HIV), Tuberculosis (TB) and malaria. Since 2002 the global fund has raised and invested more than US$55.4 billion, saving 50 million lives and reducing the combined death rate from the three diseases by more than half in the countries who they fund.^3^

The Universal Health Coverage Partnership, an initiative also of WHO is an international initiative on universal health coverage and primary care and works with several collaborators within and outside the health sector. The total amount of donor funding for this partnership is over US$300 million.

By comparison, the HEARTS partnership is unique in not being “driven” by large-scale funding. In contrast to the other partnerships, it provides technical support to scale up implementation of hypertension prevention, treatment, and control. It also facilitates strengthening of the healthcare delivery system for chronic disease management, and reorganization and reorientation of care for high blood pressure in host countries.

### Progress of HEARTS implementation

Despite only moderate investment from donor and external funding, since 2018, the WHO-HEARTS technical package has been adopted in more than 30 low- or middle-income countries across the world. The HEARTS technical package integrates key tenets of NCD care into primary care systems. Hypertension treatment is cost-effective across different LMICs (8), and the HEARTS package for addressing hypertension provides a scalable model for an important piece of the primary care puzzle in low-resource settings.

Over its first five years, the HEARTS program has demonstrated the feasibility of adopting simple treatment protocols, ensuring an adequate and reliable supply of antihypertensive medicines, training and deploying hypertension management teams, delivering patient-centered hypertension services, and standing up robust information systems for monitoring patients and programs (9). Through these programs, more than 8.7 million were initiated on treatment, and over one-third of these (36%) had documented systolic blood pressure control to <140 mm Hg as of September 1^st^, 2022. Some countries are demonstrating HTN control as high as 55–60% sub-nationally and >80% in highest performing facilities (10). Despite this early progress, the global population coverage of HEARTS remains modest.

The introduction of HEARTS in most countries have also led to major policy changes including integration into universal health coverage benefit packages, advocating for service fee capitation models as part of insurance mechanisms related to Universal Health Coverage (UHC) in countries, regulation of antihypertensives in countries, adoption of simple protocols into national guidelines, building information systems capable of capturing patient outcome data for hypertension and decentralizing care amongst healthcare professionals resulting in task shifting and team based care.

The Philippines passed the Universal Health Care Act in 2019 and uses an insurance mechanism requiring accreditation of facilities (for reimbursement) as the main way to fund UHC.^4^ The introduction of the healthy hearts project led to facilities in Iloilo in the province of Western Visayas being granted temporary accreditation for reimbursement whilst they worked on parameters to receive full accreditation. This allowed for pooling of resources and a buffer to be able to procure medication at the local level.

The HEARTS in the Americas Initiative, the largest regional contextualization of HEARTS has been implemented over the past 6 years and now includes 22 countries and 1380 facilities and are now calling for further scaling up (11).

In Vietnam the introduction of HEARTS led to a complete overhaul of the delivery of services with a shift in policy from curative services (hospitals) to preventive (public health) services at national level taking over hypertension treatment. Now patients can receive care for their hypertension at the commune health stations (community level) instead of the provincial or national outpatient departments.

The India hypertension management initiative was initially rolled out in five states between 2018 and 2019. The project has demonstrated the feasibility of implementing HEARTS even in large populations and by 2020 overall community control rates of hypertension in these states had risen from 1.4 to 5% (12).

## Discussion

**Figure 2:**
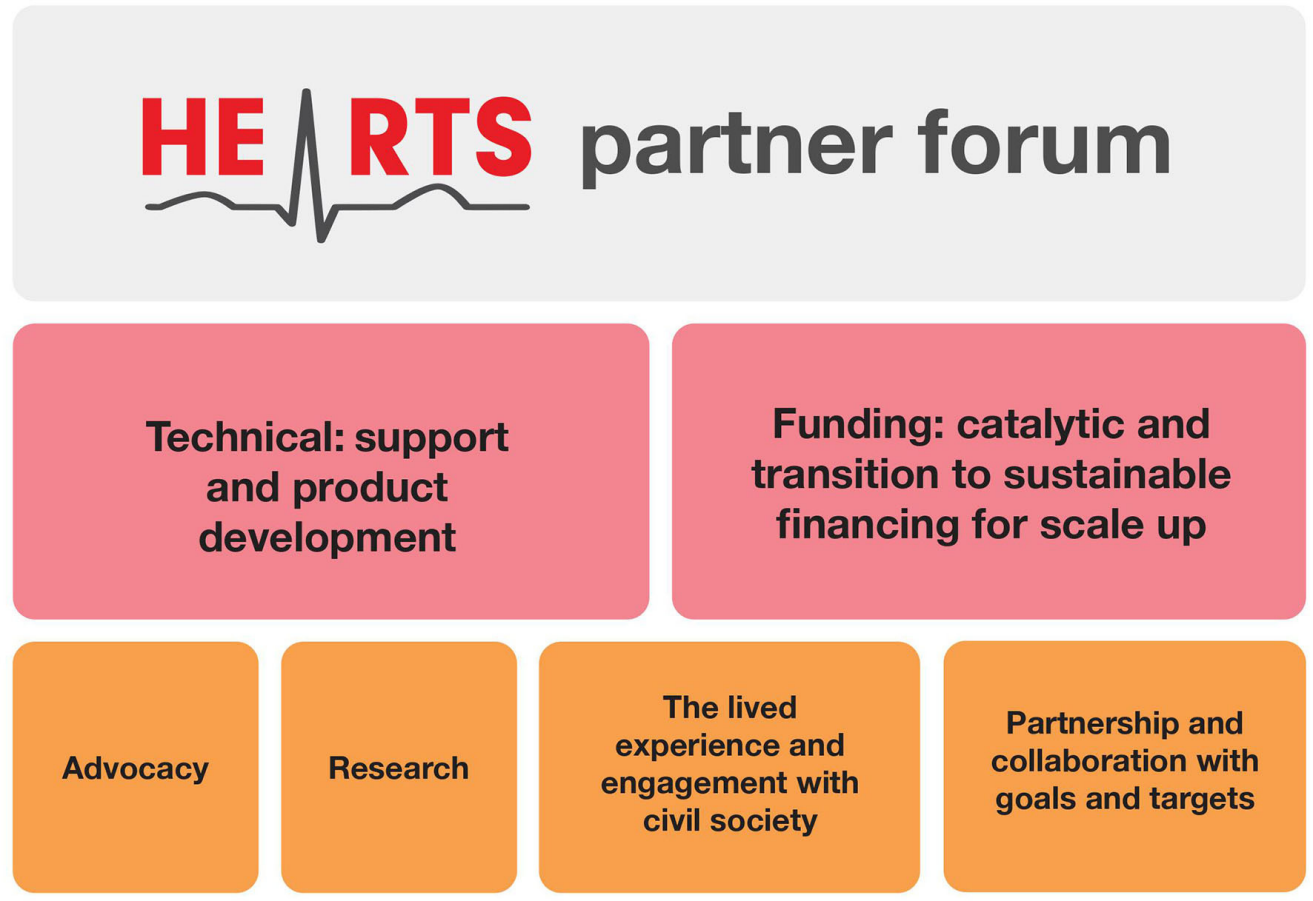
HEARTS partner forum: concept and future direction.

The HEARTS partners have been working together for 5 years. Figure 2 summarizes the concept of collaboration and outlines future directions of the partnership.

### Hypertension treatment targets

In the introduction we have explained the importance of CVD interventions to meeting SDG 3.4 with a key intervention being the improvement of hypertension control. An 80-80-80 goal for hypertension −80% of people living with hypertension (SBP ≥140 mm Hg, DBP ≥90 mm Hg, or taking antihypertensive medication) aware of their condition; 80% of those aware being treated with antihypertensive medication; 80% of those treated with their blood pressure controlled to <140/90 mmHg—resulting in an overall SBP/DBP control rate of <140/90 mm Hg of >50% in the general population will be a key strategy. A similar 90-90-90 outcome goal for HIV has yielded significant progress (13). To reach >80% awareness, the first step is to optimize opportunistic screening in all health care facilities, where undiagnosed HTN patients are “hiding in plain sight,” followed by community-based screening, if persons with probable hypertension can be reliably linked to the healthcare system. More challenges exist in countries with limited access to healthcare or where people don't visit health facilities regularly. More than 80% treatment will require systems to ensure linkage to care, relying on community outreach by lay health workers, digital hypertension registries including contact information, and a reliable supply of affordable and effective medications. Finally, reaching >80% blood pressure control means that the same delivery system includes a simple treatment protocol (including combination therapies) and health worker incentives to accelerate blood-pressure lowering and minimize therapeutic inertia. Sustained blood pressure control requires a patient-centered approach that minimizes costs to patients and delivers blood pressure management and medicines in the community near the patient's home. Implementing HEARTS successfully will require countries and health systems to set specific and progressive goals for quality of care, team-based care, medication access, good BP measurement training and certification using automated/semi-automated clinically validated oscillometric BP measurement devices, information system standards, and affordability. Future updates to HEARTS will include new modules focused on quality improvement and health economic assessment.

### Technical guidance and other implementation initiatives aligned with HEARTS

The WHO *Guideline for the pharmacological treatment of hypertension in adults* was published in 2021 (14, 15). In 2019 fixed-dose combinations for the treatment of hypertension were added to the WHO Essential Medicines List (EML) (16, 17) and guidance on technical specifications for BP devices were launched in 2021 by the WHO (18, 19). PAHO was successful in incorporating the single pill combination into the PAHO strategic fund once the combination was listed on the WHO Essential Medicine List (EML). Several parallel documents were developed by partners, such as the WHF *Roadmap for Hypertension*. The WHF roadmap for hypertension includes concrete health system and policy strategies recommendations such as community awareness campaigns, task shifting, combination therapy and digital technology strategies. The WHF roadmap also provides concrete examples and includes follow up with national roundtables (policy dialogues) to prioritize, according to the local context, which problems (and solutions) are more relevant.^5^ In 2022 a partnership between Resolve to Save Lives and WHF (with its national members) is conducting five hypertension roundtables in sub-Saharan Africa (Ghana, Uganda, Mozambique, Senegal and Cameroon), which will strengthen the implementation of HEARTS and hypertension control in sub-Saharan Africa. HEARTS in the Americas has recommended actions to catalyze full implementation of the 2021 WHO Hypertension Guidelines, including a clinical pathway that systematizes all the recommendations in this guideline to be implementing at primary health settings (20).

To further support the implementation and scale-up of HEARTS in countries, the US CDC and partners developed a costing tool for estimating the costs to run the HEARTS program from the health systems perspective. The HEARTS costing tool provides a standardized template for assessing the resources needed for HEARTS activities, including medications, training, and staff time.^6^ Health experts from several countries have employed the tool to understand the budgetary needs for the program, as well as differences between the costs of usual care and the HEARTS-recommended approach (21–23).

It has been 5 years since the HEARTS package was launched and during the implementation process many lessons have been learned regarding best practices for treatment of hypertension in LMIC primary health care facilities. For instance, HEARTS in the Americas found that common implementation barriers include segmentation of health systems, overcoming health care professionals' scope of practice legal restrictions, and lack of health information systems limiting operational evaluation and quality improvement mechanisms. Main implementation facilitators include political support from ministries of health and leading scientific societies, WHO/PAHO's role as a regional catalyst to implementation, stakeholder endorsement demonstrated by incorporating HEARTS into official documents, and having a health system oriented to primary health care (24). The resulting practical knowledge and adaptive innovations warrant an update of the HEARTS package which will be carried out over the next 2 years.

## Research

The WHO guidelines identified gaps for future areas of research. Building on that and with the support of HEARTS partners WHO is developing a document on research prioritization to improve effectiveness and efficiency of care delivery for hypertension in primary health care settings. The main objective of the document is to identify the highest priority research topics with the potential to facilitate and accelerate control. This document will have two purposes: (1) focus on key areas of research that will have direct implications on improving the management of and increase coverage of effective quality services and (2) inform funders of optimal resource allocation in program implementation.

Although the HEARTS initiative will be of central importance for improving control worldwide, it will not succeed without overcoming serious challenges. It is essential that an implementation research agenda is established in parallel with ongoing program implementation in order to study barriers to hypertension control and test innovative solutions. Implementation research will help identify the challenges related to the roll-out of the HEARTS initiative, will facilitate a better understanding of why and how the HEARTS initiative works (or does not work) in real world settings. Most importantly research findings will help in developing and testing effective strategies to overcome these challenges and introduce HEARTS into local health system at a large scale in a sustainable way. Along with formal research, less formalized continuous quality improvement programs can spur progress in hypertension and diabetes control by responding to program data in rapid intervention and feedback cycles. Resolve to Save Lives supports a global Quality Improvement Leaders Academy that provides tools and trainings for country hypertension control quality improvement programs.

HEARTS partners such as the US CDC and WHL provide a multi-part online scientific writing training course for potential investigators from low- and middle-income countries, direct mentorship for a smaller number of young professionals who are conduct of HEARTS-related research, and for generation of manuscripts that are competitive for publication in internationally recognized peer-reviewed journals.

The US CDC also supports the professional development of Field Epidemiology Training Program (FETP) residents with a portfolio of online courses to improve scientific writing skills, and self-study courses to support epidemiology, surveillance, and data analysis competencies to address CVD and CVD risk factors. CDC also supports FETP residents and recent graduates to conduct projects through the competitive Cardiovascular Small Grants Program, which helps trainees conduct clinical and community projects aligned with the strategies outlined in the HEARTS Technical Package.^7^

### Supporting expansion and scale up in countries

Most significantly, HEARTS partners will continue to support scale up and expansion in countries with a focus on Bangladesh, China, Ethiopia, India, Nigeria, Philippines, Sri Lanka, Thailand and Vietnam as well as the HEARTS in the Americas Initiative.

Innovative ways of working and collaboration amongst partners including tapping into networks of different in country organizations will help support and strengthen public health tenets that HEARTS is built on.

### Communication and advocacy

The HEARTS partner forum continues to organize activities related to communication and advocacy building greater synergy and holding greater accountability to implementation of HEARTS. Joint activities such as webinars, symposia at international meetings, including the 2022 European Society of Hypertension meeting in Athens, the International Society of Hypertension meeting in Kyoto, and American Heart Association Scientific Sessions in Chicago, the World Congress of Cardiology in Rio de Janeiro, the World Stroke Congress in Singapore, and in recognition of World Hypertension Day (May 13^th^, 2022), including the WHL World Hypertension Congress in Macau, a recent field visit and advocacy event celebrating World Heart Day (29^th^ September 2022) in a HEARTS implementation site (Iloilo, Philippines) with attendance by WHO, RTSL and WHF are examples of innovative thinking to leverage and maximize collective efforts of the partner forum.

Formalization and a structured approach to meetings, communication and dissemination are other planned activities for the future.

## Conclusion

The global scale and quality required to reach the global target of >50% population-based control will only be achieved with major changes in health care policies, reorganization of service delivery systems, training and education, financing, and practices in every HEARTS country. Likewise, a vibrant and wellorganized civil society is key for advocacy and accountability, essential principles of good governance (25).

The real question for the forum to try to answer is what will it take for HEARTS to graduate from proof-of-concept to institutionalization as standard practice in LMICs? Most necessary at the country level is country ownership of HEARTS control programs: the political will to support and sustain HEARTS with adequate planning, staffing, and financing over the long term, even when governments change. Successful HEARTS implementation will require primary health care infrastructure including services delivered to the community level, development of management skills in multiple cadres of the health care workforce, access to affordable, quality antihypertensive medicines, and a robust digital health information system that tracks standard HEARTS hypertension indicators. Simply put, HEARTS hypertension control programs form part of the foundation of each country's primary health care and the package of basic medical care that every citizen deserves. The HEARTS partner forum will be instrumental in supporting this graduation and is changing the landscape of partnerships in CVDs bringing together scientific partners, donors and public health agencies.

## Author contributions

TK conceptualized, wrote paragraphs, and was the final editor for the article. AM, PB, PW, MB, VF, DK, PR, DL, PO, AH, and DP wrote paragraphs contributing to the article. SM, BW, and RG edited the document. SS and BM provided insight and strategic direction for the article. LH assisted with overall review and editing and responses to reviewers. All authors contributed to the article and approved the submitted version.

## Footnotes

^1^ https://www.who.int/news/item/15-09-2016-global-hearts-initiative

^2^ https://www.who.int/teams/noncommunicable-diseases/ncdsmanagement/cardiovascular-diseases-programme/

^3^ https://www.theglobalfund.org/en/about-the-global-fund/

^4^ https://hta.doh.gov.ph/universal-health-care-act/#:~:text=11223%2C%20also%20known%20as%20theand%20the%20determination%20of%20a

^5^ https://globalheartjournal.com/articles/10.5334/gh.1066/

^6^ https://www.tephinet.org/tephinet-learning-center/tephinet-library/hearts-costing-tool

^7^ https://www.cdc.gov/globalhealth/healthprotection/ncd/training/hypertension-management-training.html
